# 9-*n*-Butyl-9,9′-bi[9*H*-fluorene]

**DOI:** 10.1107/S1600536808001517

**Published:** 2008-01-23

**Authors:** Shu-Qiang Yu, Bin-Bin Hu, Ping Lu

**Affiliations:** a Department of Chemistry, Zhejiang University, Yuquan Campus, Hangzhou, 310027, People’s Republic of China

## Abstract

In the title compound, C_30_H_26_, the dihedral angle between the two fluorene ring systems is 61.75 (4)°.

## Related literature

For general background, see: Muller *et al.* (2003 ▶); Murahashi & Moritani (1967 ▶).
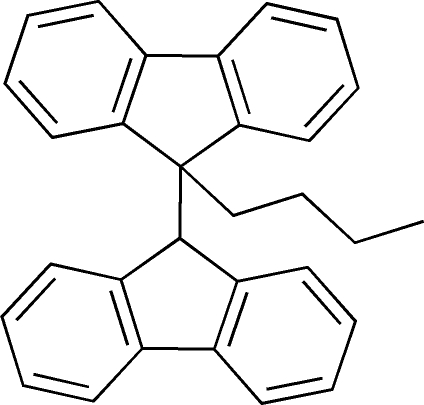


## Experimental

### 

#### Crystal data

C_30_H_26_
*M* *_r_* = 386.51
Monoclinic, 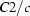

*a* = 27.164 (5) Å
*b* = 8.6369 (17) Å
*c* = 19.232 (4) Å
β = 104.28 (3)°
*V* = 4372.7 (15) Å^3^
*Z* = 8
Mo *K*α radiation
μ = 0.07 mm^−1^
*T* = 298 (2) K
0.46 × 0.38 × 0.35 mm

#### Data collection

Bruker SMART 1000 CCD area-detector diffractometer
Absorption correction: none
20365 measured reflections
4970 independent reflections
2859 reflections with *I* > 2σ(*I*)
*R*_int_ = 0.031

#### Refinement

*R*[*F* ^2^ > 2σ(*F* ^2^)] = 0.039
*wR*(*F* ^2^) = 0.122
*S* = 1.06
4970 reflections
273 parameters
H-atom parameters constrained
Δρ_max_ = 0.18 e Å^−3^
Δρ_min_ = −0.14 e Å^−3^

### 

Data collection: *SMART* (Bruker, 2001 ▶); cell refinement: *SAINT* (Bruker, 2001 ▶); data reduction: *SAINT*; program(s) used to solve structure: *SHELXTL* (Sheldrick, 2008 ▶); program(s) used to refine structure: *SHELXTL*; molecular graphics: *SHELXTL*; software used to prepare material for publication: *SHELXTL* and *publCIF* (Westrip, 2008 ▶).

## Supplementary Material

Crystal structure: contains datablocks global, I. DOI: 10.1107/S1600536808001517/xu2394sup1.cif

Structure factors: contains datablocks I. DOI: 10.1107/S1600536808001517/xu2394Isup2.hkl

Additional supplementary materials:  crystallographic information; 3D view; checkCIF report

## Figures and Tables

**Fig. 1.**
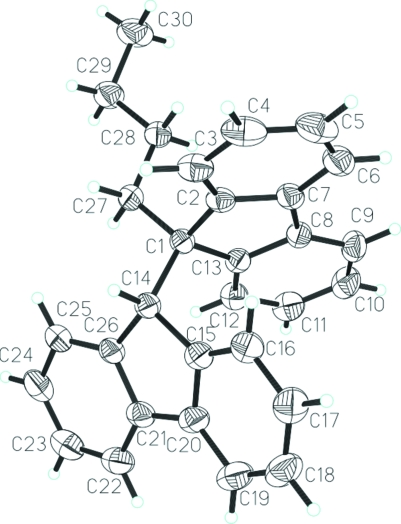
The molecular structure showing the atom-numbering scheme. Displacement ellipsoids are drawn at the 30% probability level. H atoms are shown as small spheres of arbitrary radii.

**Fig. 2.**
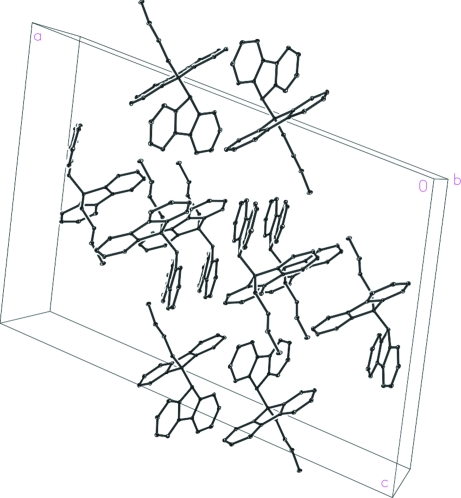
Partial packing view, H atoms are omitted for clarity.

## supplementary crystallographic information

### Comment

Fluorene derivatives, including polyfluorenes and oligofluorenes, remain a subject of intense investigation in recent years because they are very promising candidates for blue light-emitting materials in organic light-emitting devices (Muller *et al.*, 2003). The title compound, 9-*n*-butyl-9,9'-bi(9*H*-fluorene)(hereinafter abbreviated to bbf), is one of bifluorene derivatives (Murahashi & Moritani, 1967).

The asymmetric unit of the title compound contains only one bbf molecule (Fig. 1). Two fluorene rings are linked together through their 9-position carbon atoms (C1 and C14). The dihedral angle between the two fluorene rings is 61.75 (4)°. The centroid to centroid distance between stacked fluorene rings is *ca* 5.92 Å, which is very long and prevents π-π stacking (Fig. 2). All bond lengths and angles are normal.

### Experimental

All chemicals were of reagent grade quality obtained from commercial sources and used as received, unless stated otherwise. n-Butylithium (8 ml, 2.5 *M*, 20 mmol) was added to fluorene (1.66 g, 10 mmol) in 40 ml dry tetrahydrofuran under nitrogen at -78 °C. Subsequently, BF_3_Et_2_O (0.21 g, 2 mmol) was added. Kept it for 1 h, and warmed it to room temperature and stirred it overnight. After reaction completion, solvent was evaporated under reduced pressure. The crude products were purified by column chromatography (silica gel) using n-hexane/dichloromethane as eluent. The title compound was obtained as white solid in 30% yield. Colorless single crystals were grown from slow evaporation of a saturated CH_2_Cl_2_ solution of the compound.

### Refinement

H atoms were positioned geometrically and treated as riding, with C—H = 0.93 (aromatic), 0.97 (methylene) or 0.96 Å (methyl), *U*_iso_(H) = 1.5*U*_eq_(C) for methyl or *U*_iso_(H) = 1.2*U*_eq_(C) for others.

### Crystal data

**Table:**

C_30_H_26_	*F*_000_ = 1648
*M**_r_* = 386.51	*D*_x_ = 1.174 Mg m^−^^3^
Monoclinic, *C*2/*c*	Mo *K*α radiation λ = 0.71073 Å
Hall symbol: -C 2yc	Cell parameters from 12421 reflections
*a* = 27.164 (5) Å	θ = 3.0–27.4º
*b* = 8.6369 (17) Å	µ = 0.07 mm^−^^1^
*c* = 19.232 (4) Å	*T* = 298 (2) K
β = 104.28 (3)º	Chunk, colorless
*V* = 4372.7 (15) Å^3^	0.46 × 0.38 × 0.35 mm
*Z* = 8	

### Data collection

**Table:**

Bruker SMART 1000 CCD area-detector diffractometer	2859 reflections with *I* > 2σ(*I*)
Radiation source: fine-focus sealed tube	*R*_int_ = 0.031
Monochromator: graphite	θ_max_ = 27.4º
*T* = 298(2) K	θ_min_ = 3.1º
φ and ω scans	*h* = −35→35
Absorption correction: none	*k* = −11→10
20365 measured reflections	*l* = −24→24
4970 independent reflections	

### Refinement

**Table:**

Refinement on *F*^2^	Hydrogen site location: inferred from neighbouring sites
Least-squares matrix: full	H-atom parameters constrained
*R*[*F*^2^ > 2σ(*F*^2^)] = 0.039	*w* = 1/[σ^2^(*F*_o_^2^) + (0.0595*P*)^2^ + 0.3188*P*] where *P* = (*F*_o_^2^ + 2*F*_c_^2^)/3
*wR*(*F*^2^) = 0.122	(Δ/σ)_max_ < 0.001
*S* = 1.06	Δρ_max_ = 0.18 e Å^−^^3^
4970 reflections	Δρ_min_ = −0.14 e Å^−^^3^
273 parameters	Extinction correction: SHELXL, Fc^*^=kFc[1+0.001xFc^2^λ^3^/sin(2θ)]^-1/4^
Primary atom site location: structure-invariant direct methods	Extinction coefficient: 0.0038 (4)
Secondary atom site location: difference Fourier map	

### Special details

**Table:**

Experimental. ^1^H NMR (500 MHz, δ in p.p.m., CDCl_3_): 0.68-0.74 (m, 5H), 0.17-1.22 (m, 2H), 2.55-2.58 (m, 2H), 4.58 (s, 1H), 6.76 (d, 2H, *J* = 7.00 Hz), 6.86 (d, 2H, *J* = 7.00 Hz), 6.97 (t, 2H, *J* = 7.50 Hz), 7.08 (t, 2H, *J* = 7.50 Hz), 7.17-7.23 (m, 4H), 7.49 (d, 2H, *J* = 7.50 Hz); ^13^C NMR (125 MHz, δ in p.p.m., CDCl_3_): 14.15, 23.35, 26.45, 38.86, 55.48, 57.75, 119.39,119.62, 123.40, 125.84, 126.00, 126.90, 127.35, 141.59, 142.19, 144.37, 148.57; MS (EI): calcd for C_30_H_26_, 386.2; found, 386 (M^+^), 326, 313, 300, 221 (100), 179, 165, 152.
Geometry. All e.s.d.'s (except the e.s.d. in the dihedral angle between two l.s. planes) are estimated using the full covariance matrix. The cell e.s.d.'s are taken into account individually in the estimation of e.s.d.'s in distances, angles and torsion angles; correlations between e.s.d.'s in cell parameters are only used when they are defined by crystal symmetry. An approximate (isotropic) treatment of cell e.s.d.'s is used for estimating e.s.d.'s involving l.s. planes.
Refinement. Refinement of *F*^2^ against ALL reflections. The weighted *R*-factor *wR* and goodness of fit *S* are based on *F*^2^, conventional *R*-factors *R* are based on *F*, with *F* set to zero for negative *F*^2^. The threshold expression of *F*^2^ > σ(*F*^2^) is used only for calculating *R*-factors(gt) *etc*. and is not relevant to the choice of reflections for refinement. *R*-factors based on *F*^2^ are statistically about twice as large as those based on *F*, and *R*- factors based on ALL data will be even larger.

### Fractional atomic coordinates and isotropic or equivalent isotropic displacement parameters (Å^2^)

**Table:**

	*x*	*y*	*z*	*U*_iso_*/*U*_eq_	
C1	0.11948 (5)	0.49033 (14)	−0.00529 (6)	0.0446 (3)	
C2	0.09626 (5)	0.61937 (15)	−0.05656 (7)	0.0482 (3)	
C3	0.05151 (6)	0.61827 (19)	−0.11011 (7)	0.0622 (4)	
H3	0.0322	0.5285	−0.1203	0.075*	
C4	0.03611 (7)	0.7533 (2)	−0.14825 (8)	0.0765 (5)	
H4	0.0058	0.7547	−0.1836	0.092*	
C5	0.06533 (8)	0.8858 (2)	−0.13425 (9)	0.0788 (5)	
H5	0.0546	0.9751	−0.1607	0.095*	
C6	0.11021 (7)	0.88774 (17)	−0.08167 (9)	0.0669 (4)	
H6	0.1299	0.9770	−0.0728	0.080*	
C7	0.12544 (5)	0.75404 (15)	−0.04213 (7)	0.0501 (3)	
C8	0.16888 (5)	0.72391 (15)	0.01911 (7)	0.0492 (3)	
C9	0.20847 (6)	0.81905 (18)	0.05433 (9)	0.0648 (4)	
H9	0.2109	0.9204	0.0392	0.078*	
C10	0.24415 (6)	0.7601 (2)	0.11223 (9)	0.0730 (5)	
H10	0.2707	0.8227	0.1365	0.088*	
C11	0.24097 (6)	0.6098 (2)	0.13449 (8)	0.0679 (4)	
H11	0.2653	0.5725	0.1737	0.082*	
C12	0.20198 (5)	0.51323 (17)	0.09921 (7)	0.0562 (4)	
H12	0.2003	0.4113	0.1141	0.067*	
C13	0.16563 (5)	0.57100 (14)	0.04155 (6)	0.0453 (3)	
C14	0.08081 (5)	0.43871 (14)	0.03910 (7)	0.0481 (3)	
H14	0.0498	0.4014	0.0055	0.058*	
C15	0.06633 (5)	0.56906 (16)	0.08363 (7)	0.0518 (3)	
C16	0.04276 (5)	0.71006 (18)	0.06246 (9)	0.0617 (4)	
H16	0.0342	0.7384	0.0143	0.074*	
C17	0.03222 (6)	0.8079 (2)	0.11398 (10)	0.0757 (5)	
H17	0.0167	0.9028	0.1002	0.091*	
C18	0.04449 (7)	0.7664 (2)	0.18550 (10)	0.0828 (5)	
H18	0.0371	0.8337	0.2193	0.099*	
C19	0.06752 (7)	0.6270 (2)	0.20742 (9)	0.0744 (5)	
H19	0.0755	0.5993	0.2556	0.089*	
C20	0.07864 (5)	0.52777 (18)	0.15625 (7)	0.0566 (4)	
C21	0.09993 (5)	0.37098 (17)	0.16385 (7)	0.0553 (4)	
C22	0.11628 (6)	0.2786 (2)	0.22436 (8)	0.0694 (4)	
H22	0.1170	0.3176	0.2697	0.083*	
C23	0.13134 (7)	0.1289 (2)	0.21644 (10)	0.0774 (5)	
H23	0.1429	0.0670	0.2567	0.093*	
C24	0.12937 (7)	0.07014 (19)	0.14925 (10)	0.0768 (5)	
H24	0.1392	−0.0317	0.1446	0.092*	
C25	0.11283 (6)	0.16108 (17)	0.08828 (8)	0.0661 (4)	
H25	0.1109	0.1196	0.0430	0.079*	
C26	0.09925 (5)	0.31399 (16)	0.09538 (7)	0.0519 (3)	
C27	0.13491 (5)	0.35210 (15)	−0.04660 (7)	0.0525 (3)	
H27A	0.1531	0.2773	−0.0121	0.063*	
H27B	0.1043	0.3022	−0.0742	0.063*	
C28	0.16785 (6)	0.39514 (15)	−0.09723 (7)	0.0558 (4)	
H28A	0.1501	0.4721	−0.1310	0.067*	
H28B	0.1990	0.4421	−0.0695	0.067*	
C29	0.18128 (7)	0.26016 (17)	−0.13865 (8)	0.0685 (4)	
H29A	0.1503	0.2082	−0.1635	0.082*	
H29B	0.2014	0.1869	−0.1052	0.082*	
C30	0.21068 (8)	0.3067 (2)	−0.19310 (9)	0.0881 (6)	
H30A	0.1908	0.3783	−0.2269	0.132*	
H30B	0.2177	0.2164	−0.2181	0.132*	
H30C	0.2420	0.3550	−0.1688	0.132*	

### Atomic displacement parameters (Å^2^)

**Table:**

	*U*^11^	*U*^22^	*U*^33^	*U*^12^	*U*^13^	*U*^23^
C1	0.0415 (7)	0.0482 (7)	0.0460 (7)	−0.0060 (6)	0.0142 (5)	−0.0020 (6)
C2	0.0433 (7)	0.0593 (8)	0.0459 (7)	−0.0001 (6)	0.0186 (6)	0.0001 (6)
C3	0.0508 (9)	0.0868 (11)	0.0499 (8)	0.0004 (8)	0.0139 (7)	0.0039 (8)
C4	0.0623 (11)	0.1144 (15)	0.0535 (9)	0.0212 (10)	0.0157 (8)	0.0162 (10)
C5	0.0905 (14)	0.0866 (12)	0.0679 (10)	0.0336 (11)	0.0358 (10)	0.0248 (10)
C6	0.0836 (12)	0.0559 (8)	0.0704 (10)	0.0098 (8)	0.0365 (9)	0.0072 (8)
C7	0.0529 (8)	0.0493 (8)	0.0549 (8)	0.0031 (6)	0.0260 (6)	0.0012 (6)
C8	0.0473 (8)	0.0491 (7)	0.0567 (8)	−0.0060 (6)	0.0232 (6)	−0.0071 (6)
C9	0.0626 (10)	0.0559 (8)	0.0801 (10)	−0.0169 (7)	0.0258 (8)	−0.0134 (8)
C10	0.0554 (10)	0.0829 (12)	0.0798 (11)	−0.0220 (8)	0.0146 (9)	−0.0215 (9)
C11	0.0441 (9)	0.0922 (12)	0.0640 (9)	−0.0055 (8)	0.0068 (7)	−0.0079 (9)
C12	0.0448 (8)	0.0635 (8)	0.0600 (8)	−0.0021 (7)	0.0123 (6)	0.0001 (7)
C13	0.0383 (7)	0.0513 (7)	0.0494 (7)	−0.0032 (6)	0.0169 (6)	−0.0062 (6)
C14	0.0437 (7)	0.0556 (7)	0.0470 (7)	−0.0109 (6)	0.0148 (6)	−0.0044 (6)
C15	0.0368 (7)	0.0645 (8)	0.0575 (8)	−0.0096 (6)	0.0182 (6)	−0.0080 (7)
C16	0.0473 (8)	0.0704 (9)	0.0729 (9)	−0.0019 (7)	0.0254 (7)	−0.0040 (8)
C17	0.0625 (11)	0.0751 (11)	0.0997 (13)	0.0026 (8)	0.0394 (9)	−0.0123 (10)
C18	0.0761 (12)	0.0938 (13)	0.0885 (13)	−0.0032 (10)	0.0390 (10)	−0.0320 (11)
C19	0.0682 (11)	0.0985 (13)	0.0610 (9)	−0.0059 (10)	0.0248 (8)	−0.0185 (9)
C20	0.0422 (8)	0.0766 (10)	0.0537 (8)	−0.0100 (7)	0.0172 (6)	−0.0111 (7)
C21	0.0441 (8)	0.0723 (9)	0.0510 (8)	−0.0129 (7)	0.0146 (6)	−0.0014 (7)
C22	0.0606 (10)	0.0949 (12)	0.0529 (8)	−0.0117 (9)	0.0147 (7)	0.0030 (9)
C23	0.0701 (11)	0.0909 (12)	0.0723 (11)	−0.0040 (9)	0.0199 (9)	0.0229 (10)
C24	0.0811 (12)	0.0638 (10)	0.0934 (13)	−0.0049 (9)	0.0365 (10)	0.0171 (10)
C25	0.0790 (11)	0.0602 (9)	0.0667 (9)	−0.0149 (8)	0.0325 (8)	0.0007 (8)
C26	0.0475 (8)	0.0594 (8)	0.0524 (8)	−0.0146 (6)	0.0191 (6)	−0.0021 (7)
C27	0.0569 (9)	0.0514 (7)	0.0531 (7)	−0.0082 (6)	0.0208 (6)	−0.0051 (6)
C28	0.0611 (9)	0.0551 (8)	0.0562 (8)	−0.0035 (7)	0.0242 (7)	−0.0029 (7)
C29	0.0848 (12)	0.0617 (9)	0.0693 (10)	0.0068 (8)	0.0389 (9)	−0.0003 (8)
C30	0.1143 (16)	0.0845 (12)	0.0849 (12)	0.0179 (11)	0.0617 (11)	0.0060 (10)

### Geometric parameters (Å, °)

**Table:**

C1—C2	1.5183 (18)	C16—H16	0.9300
C1—C13	1.5203 (17)	C17—C18	1.380 (2)
C1—C27	1.5481 (18)	C17—H17	0.9300
C1—C14	1.5728 (18)	C18—C19	1.374 (2)
C2—C3	1.386 (2)	C18—H18	0.9300
C2—C7	1.3963 (18)	C19—C20	1.393 (2)
C3—C4	1.386 (2)	C19—H19	0.9300
C3—H3	0.9300	C20—C21	1.466 (2)
C4—C5	1.381 (2)	C21—C22	1.390 (2)
C4—H4	0.9300	C21—C26	1.4018 (18)
C5—C6	1.379 (3)	C22—C23	1.376 (2)
C5—H5	0.9300	C22—H22	0.9300
C6—C7	1.3884 (19)	C23—C24	1.377 (2)
C6—H6	0.9300	C23—H23	0.9300
C7—C8	1.470 (2)	C24—C25	1.391 (2)
C8—C9	1.3897 (19)	C24—H24	0.9300
C8—C13	1.3989 (18)	C25—C26	1.387 (2)
C9—C10	1.381 (2)	C25—H25	0.9300
C9—H9	0.9300	C27—C28	1.5222 (19)
C10—C11	1.376 (2)	C27—H27A	0.9700
C10—H10	0.9300	C27—H27B	0.9700
C11—C12	1.387 (2)	C28—C29	1.5067 (19)
C11—H11	0.9300	C28—H28A	0.9700
C12—C13	1.3833 (19)	C28—H28B	0.9700
C12—H12	0.9300	C29—C30	1.520 (2)
C14—C26	1.5217 (19)	C29—H29A	0.9700
C14—C15	1.5237 (18)	C29—H29B	0.9700
C14—H14	0.9800	C30—H30A	0.9600
C15—C16	1.389 (2)	C30—H30B	0.9600
C15—C20	1.3997 (19)	C30—H30C	0.9600
C16—C17	1.385 (2)		
			
C2—C1—C13	101.43 (10)	C18—C17—C16	120.85 (17)
C2—C1—C27	110.77 (10)	C18—C17—H17	119.6
C13—C1—C27	111.68 (11)	C16—C17—H17	119.6
C2—C1—C14	109.53 (10)	C19—C18—C17	120.85 (16)
C13—C1—C14	111.83 (10)	C19—C18—H18	119.6
C27—C1—C14	111.19 (10)	C17—C18—H18	119.6
C3—C2—C7	120.28 (13)	C18—C19—C20	118.94 (16)
C3—C2—C1	128.70 (13)	C18—C19—H19	120.5
C7—C2—C1	110.96 (11)	C20—C19—H19	120.5
C2—C3—C4	118.83 (15)	C19—C20—C15	120.53 (15)
C2—C3—H3	120.6	C19—C20—C21	130.31 (14)
C4—C3—H3	120.6	C15—C20—C21	109.04 (12)
C5—C4—C3	120.68 (16)	C22—C21—C26	120.82 (15)
C5—C4—H4	119.7	C22—C21—C20	130.58 (14)
C3—C4—H4	119.7	C26—C21—C20	108.50 (12)
C6—C5—C4	120.98 (15)	C23—C22—C21	119.27 (15)
C6—C5—H5	119.5	C23—C22—H22	120.4
C4—C5—H5	119.5	C21—C22—H22	120.4
C5—C6—C7	118.81 (16)	C22—C23—C24	120.43 (16)
C5—C6—H6	120.6	C22—C23—H23	119.8
C7—C6—H6	120.6	C24—C23—H23	119.8
C6—C7—C2	120.40 (14)	C23—C24—C25	120.85 (17)
C6—C7—C8	131.13 (13)	C23—C24—H24	119.6
C2—C7—C8	108.42 (11)	C25—C24—H24	119.6
C9—C8—C13	120.53 (13)	C26—C25—C24	119.55 (15)
C9—C8—C7	131.11 (13)	C26—C25—H25	120.2
C13—C8—C7	108.36 (11)	C24—C25—H25	120.2
C10—C9—C8	118.67 (14)	C25—C26—C21	118.99 (13)
C10—C9—H9	120.7	C25—C26—C14	130.76 (12)
C8—C9—H9	120.7	C21—C26—C14	110.21 (12)
C11—C10—C9	120.90 (14)	C28—C27—C1	114.59 (11)
C11—C10—H10	119.5	C28—C27—H27A	108.6
C9—C10—H10	119.5	C1—C27—H27A	108.6
C10—C11—C12	120.93 (15)	C28—C27—H27B	108.6
C10—C11—H11	119.5	C1—C27—H27B	108.6
C12—C11—H11	119.5	H27A—C27—H27B	107.6
C13—C12—C11	118.90 (14)	C29—C28—C27	113.98 (11)
C13—C12—H12	120.6	C29—C28—H28A	108.8
C11—C12—H12	120.6	C27—C28—H28A	108.8
C12—C13—C8	120.06 (12)	C29—C28—H28B	108.8
C12—C13—C1	129.12 (12)	C27—C28—H28B	108.8
C8—C13—C1	110.82 (11)	H28A—C28—H28B	107.7
C26—C14—C15	102.04 (10)	C28—C29—C30	113.36 (13)
C26—C14—C1	116.10 (11)	C28—C29—H29A	108.9
C15—C14—C1	113.28 (10)	C30—C29—H29A	108.9
C26—C14—H14	108.3	C28—C29—H29B	108.9
C15—C14—H14	108.3	C30—C29—H29B	108.9
C1—C14—H14	108.3	H29A—C29—H29B	107.7
C16—C15—C20	119.68 (13)	C29—C30—H30A	109.5
C16—C15—C14	130.33 (13)	C29—C30—H30B	109.5
C20—C15—C14	109.96 (12)	H30A—C30—H30B	109.5
C17—C16—C15	119.15 (15)	C29—C30—H30C	109.5
C17—C16—H16	120.4	H30A—C30—H30C	109.5
C15—C16—H16	120.4	H30B—C30—H30C	109.5
			
C13—C1—C2—C3	−176.43 (13)	C13—C1—C14—C15	51.64 (14)
C27—C1—C2—C3	64.89 (18)	C27—C1—C14—C15	177.25 (11)
C14—C1—C2—C3	−58.12 (17)	C26—C14—C15—C16	−173.67 (13)
C13—C1—C2—C7	1.02 (13)	C1—C14—C15—C16	60.77 (18)
C27—C1—C2—C7	−117.65 (12)	C26—C14—C15—C20	4.01 (14)
C14—C1—C2—C7	119.34 (11)	C1—C14—C15—C20	−121.55 (12)
C7—C2—C3—C4	−0.8 (2)	C20—C15—C16—C17	0.5 (2)
C1—C2—C3—C4	176.49 (13)	C14—C15—C16—C17	177.98 (14)
C2—C3—C4—C5	1.4 (2)	C15—C16—C17—C18	−0.5 (2)
C3—C4—C5—C6	−0.6 (3)	C16—C17—C18—C19	0.0 (3)
C4—C5—C6—C7	−0.7 (2)	C17—C18—C19—C20	0.4 (3)
C5—C6—C7—C2	1.3 (2)	C18—C19—C20—C15	−0.3 (2)
C5—C6—C7—C8	−175.78 (14)	C18—C19—C20—C21	−175.94 (15)
C3—C2—C7—C6	−0.6 (2)	C16—C15—C20—C19	−0.1 (2)
C1—C2—C7—C6	−178.28 (12)	C14—C15—C20—C19	−178.06 (13)
C3—C2—C7—C8	177.11 (12)	C16—C15—C20—C21	176.36 (12)
C1—C2—C7—C8	−0.59 (15)	C14—C15—C20—C21	−1.60 (15)
C6—C7—C8—C9	−2.2 (3)	C19—C20—C21—C22	−1.9 (3)
C2—C7—C8—C9	−179.55 (14)	C15—C20—C21—C22	−177.95 (15)
C6—C7—C8—C13	177.20 (14)	C19—C20—C21—C26	174.22 (15)
C2—C7—C8—C13	−0.16 (15)	C15—C20—C21—C26	−1.79 (15)
C13—C8—C9—C10	−0.6 (2)	C26—C21—C22—C23	−0.8 (2)
C7—C8—C9—C10	178.69 (14)	C20—C21—C22—C23	174.97 (15)
C8—C9—C10—C11	0.5 (2)	C21—C22—C23—C24	−1.2 (3)
C9—C10—C11—C12	0.3 (3)	C22—C23—C24—C25	0.9 (3)
C10—C11—C12—C13	−0.9 (2)	C23—C24—C25—C26	1.5 (3)
C11—C12—C13—C8	0.8 (2)	C24—C25—C26—C21	−3.4 (2)
C11—C12—C13—C1	−179.59 (13)	C24—C25—C26—C14	179.11 (14)
C9—C8—C13—C12	0.0 (2)	C22—C21—C26—C25	3.1 (2)
C7—C8—C13—C12	−179.46 (12)	C20—C21—C26—C25	−173.51 (13)
C9—C8—C13—C1	−179.69 (12)	C22—C21—C26—C14	−178.93 (13)
C7—C8—C13—C1	0.84 (14)	C20—C21—C26—C14	4.46 (15)
C2—C1—C13—C12	179.22 (13)	C15—C14—C26—C25	172.52 (14)
C27—C1—C13—C12	−62.77 (17)	C1—C14—C26—C25	−63.80 (19)
C14—C1—C13—C12	62.57 (17)	C15—C14—C26—C21	−5.13 (14)
C2—C1—C13—C8	−1.12 (13)	C1—C14—C26—C21	118.54 (12)
C27—C1—C13—C8	116.89 (12)	C2—C1—C27—C28	51.73 (15)
C14—C1—C13—C8	−117.77 (12)	C13—C1—C27—C28	−60.53 (15)
C2—C1—C14—C26	−177.61 (10)	C14—C1—C27—C28	173.78 (11)
C13—C1—C14—C26	−65.98 (14)	C1—C27—C28—C29	−178.29 (13)
C27—C1—C14—C26	59.63 (14)	C27—C28—C29—C30	175.53 (14)
C2—C1—C14—C15	−59.99 (13)		
